# A One Health Evaluation of the University of Copenhagen Research Centre for Control of Antibiotic Resistance

**DOI:** 10.3389/fvets.2018.00194

**Published:** 2018-08-21

**Authors:** Anaïs Léger, Katharina D.C. Stärk, Jonathan Rushton, Liza R. Nielsen

**Affiliations:** ^1^SAFOSO AG, Bern, Switzerland; ^2^Faculty of Health and Life Science, Institute of Infection and Global Health, University of Liverpool, Liverpool, United Kingdom; ^3^Department of Veterinary and Animal Sciences, Faculty of Health and Medical Sciences, University of Copenhagen, Frederiksberg, Denmark

**Keywords:** One Health, evaluation, AMR-research, theory of change, outcomes

## Abstract

We applied the evaluation framework developed by the EU COST Action “Network of Evaluation of One Health” (NEOH) to assess the operations, supporting infrastructures and outcomes of a research consortium “University of Copenhagen Research Centre for Control of Antibiotic Resistance” (UC-CARE). This 4-year research project was a One Health (OH) initiative with participants from 14 departments over four faculties as well as stakeholders from industry and health authorities aiming to produce new knowledge to reduce the development of antimicrobial resistance (AMR). This was a case study focusing on assessing beneficial and counter-productive characteristics that could affect the OH outcomes. The study was also used to provide feedback to NEOH about the evaluation framework. The framework and evaluation tools are described in the introduction paper of this special journal issue. Data for the evaluation were extracted from the funding research proposal, the mid-term UC-CARE project evaluation report and supplemented with opinions elicited from project participants and stakeholders. Here, we describe the underlying system, theory of change behind the initiative and adapted questions from the NEOH tools that we used for semi-open interviews with consortium members throughout the evaluation process. An online survey was used to obtain information from stakeholders. The NEOH evaluation tools were then used for the qualitative and quantitative evaluation of the OH characteristics of UC-CARE. Senior UC-CARE researchers were interested and willing to be interviewed. Young scientists were more difficult to engage in interviews, and only 25% of stakeholders answered the online survey. Interviewees mentioned that the main benefit of UC-CARE was an increased awareness and general understanding of AMR issues. All interviewees stated that the adopted OH approach was relevant given the complexity of AMR. However, some questioned the applicability, and identified potentially counter-productive issues mainly related to the information sharing, collaboration and working methods across the consortium. A more integrated project organization, more stakeholder involvement and time for the project, flexibility in planning and a dedicated OH coordinator were suggested to allow for more knowledge exchange, potentially leading to a higher societal impact.

## Introduction

Antibiotics are used to treat domesticated terrestrial and aquatic animals, plants, and humans from bacterial infections, but their lack of effectiveness on some resistant bacteria has resulted in deaths and the increased suffering of people and animals (1, 2). Antimicrobial resistance (AMR) is a global issue concerning human and animal health, and several actions are being taken at national and international levels to slow down and reduce this trend (3). In the context of a growing human population, with greater domesticated animal populations, healthcare systems relying on antibiotics for their effectiveness, and international travel and trade, there is an increasing need for the improved management and use of antimicrobials and the pursuit of alternatives to existing antimicrobial compounds (1, 4).

A One Health (OH) approach is recommended by the scientific community and international organizations to solve complex situations and new issues such as AMR (2, 5–7). Researchers from different disciplines need to collaborate and seek support and involvement across multiple sectors. OH approaches have been implemented in diverse fields and the benefit has been demonstrated in different studies (8). Among the numerous examples of OH approaches (9, 10) are those relating to the surveillance of zoonotic or food-borne diseases (11–14), effective disease control policies (15) and implementation of control measures (16–19) and research (20–22).

The “University of Copenhagen Research Centre for Control of Antibiotic Resistance” (UC-CARE) was a collaborative effort across a number of disciplines. It adopted an OH approach as the central theme of the consortium, launching a large 4-year research project in 2013 (23). UC-CARE aimed to provide knowledge to combat AMR through inter-sectorial collaboration between the human and animal health sectors. It involved 14 departments across four faculties of the University of Copenhagen and engaged the Danish livestock farming and pharmaceutical industries and national health authorities as the main stakeholders. The research project had six research work packages (WPs) with specific objectives and varying numbers of researchers (WP leaders, PhD fellows, Post-docs and Assistant/Associate Professors), one WP dedicated to dissemination and education, and one management WP with a management board, stakeholder board and scientific advisory board. The initial budget in the project proposal was 34.7 million DKK, including salaries for 636 man-months (53 years) of research and 54 man-months of technical and administrative support. The initiative therefore constituted a significant OH effort, funded mainly by the university itself.

A mid-term evaluation of the UC-CARE project was conducted in May 2016 by external researchers, and a final evaluation was planned after completion of the project. However, commonly used frameworks for research project evaluations do not cover all the aspects of OH initiatives that are relevant to the outcomes and societal impacts that such initiatives are aimed toward (24). Rather, OH initiatives should be evaluated using methods targeting the transdisciplinarity of the initiative and the potential added value of choosing that approach over a less integrated approach. For this reason, a “Network for Evaluation of One Health” (NEOH) (25) was created in 2014, supported by a European Corporation in Science & Technology (EU COST) action to develop evidence-based guidelines and tools for the qualitative and quantitative assessment of OH initiatives. The developed framework and evaluation tools have been presented in detail by NEOH consortium members in the Frontiers journal research topic^1^ (26). In short, it consists of four elements: (i) a description of the underlying system and the OH initiative in relation to the system; (ii) the theory of change behind the initiative, including expected outcomes; (iii) the evaluation process supported by evaluation tools that summarize the qualitative and quantitative evaluation of operations and supporting infrastructures in the initiative (referred to as “the One Health-ness” of the initiative); (iv) a comparison of the One Health-ness and the outcomes of the initiative. The elements are described in more detail in sections Materials and Methods and The Resulting Evaluation of UC-CARE below.

During 2016 and 2017, case studies were carried out by NEOH consortium members on real life OH initiatives to assess the usefulness and present the application of the NEOH framework. Two objectives of the case study were presented: (1) to evaluate the transdisciplinarity and outcomes of the UC-CARE consortium and research project using the NEOH evaluation framework and tools, and (2) to assess the usefulness of the NEOH framework and tools for further refinement. As we decided to illustrate the results of the UC-CARE evaluation using the amended and published NEOH framework resulting from the case study feedback, the following report will focus on the first objective.

## Materials and methods

### Methods for the evaluation of UC-CARE

The evaluation mainly took place in November and December 2016 and was fully supported by the management board of UC-CARE. In October 2016, the principal investigator (PI) encouraged all actors in the consortium to agree to an interview with the external evaluator.

Defining and agreeing upon an evaluation question is important, and in this case study, the question was: which elements of UC-CARE were particularly productive and efficient, and which elements could be improved to ensure that the expected intermediary outcomes (i.e., high research quality and substantial output) will ultimately lead to a positive impact on human, animal, and environmental health, given the available resources in the consortium?

To answer the evaluation question, it is necessary for the evaluator to first develop a thorough understanding of the structures, boundaries, and dynamics of the initiative, as well as an overview of the actors involved and stakeholders affected by or interacting with the initiative. The initiative should be seen in relation to the underlying system for which it is intended have an impact (i.e., the context). This constitutes the first evaluation element in the NEOH framework (26).

The second element in the evaluation is to describe the theory of change (TOC), which is an outcome-oriented approach to describing the logics and reasoning behind the design of the initiative, including the identification of expected outputs and outcomes, arising within and between disciplines, and the resulting expected impacts on the underlying system. The TOC was defined by Rüegg et al. (26): “The TOC explains all the different pathways that might lead to the desired effect of an initiative. It not only shows the outputs, outcomes, and impact of an initiative, but also requires outlining (and explaining) the causal linkages. Each effect is shown in a logical relationship to all the others.” For the UC-CARE case study, information about the initiative and the expected impact on the underlying system as well as the TOC were obtained from the original research proposal and the mid-way evaluation report in which the consortium design, aims, outcomes and expected societal impacts were partly described, supplemented with interviews with the principle investigators and work-package (WP) leaders, who knew more about the process of the development of the consortium (we refer to section Data Collection and Methods for Assessing OH Outcomes and Outputs below for more information).

The third element is the evaluation process, supported by tools to summarize the qualitative and quantitative evaluation of operations and supporting infrastructures in the initiative (26). This is also referred to as “the OH-ness” of the initiative, and the method used is described in section Methods for Assessing the OH-ness of the Initiative below.

Finally, the fourth element is a comparison of “the OH-ness” with the outcomes of the initiative. For this particular study, this could only be addressed in a qualitative and descriptive way in the discussion of the results (section Discussion). Future comparison across multiple case studies may lead to a better understanding of the optimal attributes of OH initiatives, but this is beyond the scope of this paper.

### Data collection

Data collection was conducted over 3 weeks in November 2016. It was based on (i) face-to-face interviews with consortium partners, (ii) an online survey for stakeholders and external partners, and (iii) internal documentation of UC-CARE (i.e., the initial proposal, mid-term evaluation). The data were collected by the first author, who was the external evaluator of the UC-CARE consortium.

No ethical approval was required for this study in accordance with the national and institutional requirements. All interviewees and stakeholders participated voluntarily and remain anonymous.

#### Face-to-face interviews among the consortium

One-hour long, semi-open interviews were conducted with consortium partners selected according to their role and commitment to the project. Three PIs and deputies, six research WP leaders, 20 young researchers (PhD and Post-doc students) and four other selected key people in the consortium were all targeted for interview. The WPs are described in relation to the system in section The OH Initiative UC-CARE Within the System. The constitution of each WP varied depending on available funding over the project period. The last author contacted the targeted interviewees by email to present the evaluation purpose and introduce the interviewer. Thereafter, the first author contacted them personally to schedule the interview.

The interview questions were adapted from the identified information developed in the NEOH tools (26). At the end of every interview, participants were also asked to go back to the original proposal and provide feedback about their results and their perception of progress in the efforts against AMR. The questionnaire was pre-tested through one pilot interview to minimize question ambiguity and generally refine the process. The questionnaire is available in Annex 1 (Supplementary Material).

All interviews were recorded with a simple voice recorder, stored as MP4 audio files and then transcribed by the first author after each interview in order to have access to all valuable information provided by the interviewees.

#### Online survey for stakeholders and external partners

All 27 people on the UC-CARE mailing list for external partners, as well as members of the scientific advisory board and stakeholder board were contacted by personal email to take part in the survey by answering the online questionnaire.

The questionnaire was designed using Google Forms, and included 19 questions that took around 15–20 min to answer. The interview questions were adapted from the identified information developed in the NEOH tools (26). The questionnaire is available in Annex 2 (Supplementary Material). The questionnaire was pre-tested through two pilot interviews to minimize question ambiguity and generally refine the process.

### Methods for assessing the OH-ness of the initiative

After the data and information were collected, the OH-ness of UC-CARE was assessed by following the format developed in the NEOH tools, which was provided in the form of Microsoft Excel spreadsheets with explanations, guidelines and pre-defined calculation formulae for overall measures (26). The OH-ness is the sum of several characteristics that define integrated approaches and transdisciplinarity, including the operations: OH thinking, OH planning, and OH working, and the supporting infrastructures: OH sharing, OH learning, and systemic organization (26). A training session for future evaluators was organized by the NEOH consortium in July 2016 to promote an understanding of the tools and how to use them.

While evaluating OH thinking, we assess how well and how much the dimensions (hierarchical orders of systems' organization) covered by the initiative fit with the real context of the research topic. It therefore helps to understand whether the initiative addresses appropriate aspects of the context so as to have the desired impact on the identified health challenge. The OH planning characteristics concern the organization of tasks in relation to the resources and responsibilities needed to complete the defined tasks and objectives over the duration of the initiative. Some of the questions must be defined in the planning tool, and can therefore differ among initiatives. Evaluation of OH working characteristics focuses on the broadness of the initiative in relation to disciplines and societal involvement, collaboration and the flexibility to adjust to changing conditions during the working phase of the initiative. When assessing OH sharing, evaluators must investigate the exchange of data and information across the initiative and with stakeholders. OH learning includes knowledge and understanding of the context and the initiative outputs at individual, group and organizational level. Systemic organization is evaluated in terms of the alignment of goals, actors and competencies, as well as relevant leadership skills and behavior.

For each OH characteristic, the identified metrics to support the evaluation were organized into a framework and phrased as a question (Annex 3 in Supplementary Material). Every question was scored based on a detailed explanation with arguments for the score. Each OH characteristic was assessed by a final score, which summarized the question-specific scores. All OH characteristics are depicted on a scale from 0 to 1 as a spoke of a diagram found in the sheet named “OH diagram” (26). The diagram surface of the initiative was then calculated relative to the maximum surface attainable, thus referring to the degree of OH integration in the initiative (i.e., the OH index, which is a number between 0 and 1). The balance between operational and supporting means was extrapolated from this (i.e., the OH ratio).

The qualitative and quantitative assessment was mainly conducted by the first and last author. A general review of the evaluation and interpretation of results was completed by the two other authors. Preliminary results were also presented to the UC-CARE consortium during the last annual meeting. Comments from the audience were then integrated into the final version of the evaluation, which was sent to the UC-CARE management board for comment and to check potential misunderstandings before submission of the manuscript for publication.

### Methods for assessing OH outcomes and outputs

A TOC was derived from the UC-CARE proposal to support the identification of outcomes and outputs and illustrated in a diagram (26). Realized outputs and outcomes were identified from the online survey, interviews, internal UC-CARE documents, and comments from the PI upon the final evaluation check. Since outputs and outcomes continue to result from the UC-CARE consortium, it is worth noting that those included here were realized by July 2017.

Considered outputs and outcomes were categorized as: disciplinary, interdisciplinary, OH, and unexpected. The analysis of outputs/outcomes was based on overall numeric and textual methods. However, it was not possible to count the exact number of research publications originating from the consortium after the mid-term report, as these were not collected in a central place and due to a lack of response from many consortium participants. This highlights the information-sharing issues in UC-CARE, as discussed below.

## The resulting evaluation of UC-CARE

This section summarizes the full evaluation with regard to the four evaluation elements in the NEOH framework, i.e., the description of (i) the initiative within its identified context, (ii) the TOC, (iii) the OH-ness assessment and (iv) outcomes of the initiative.

In total, 18 consortium partners were interviewed: 10 individual interviews, two group interviews (each with two early-career researchers) and one group interview with four actors. At least one person was interviewed from each WP. It was difficult to count the number of partners in the consortium because this changed over time. However, there were 26 PhD and Post-doc fellows involved, and approximately the same number of more senior researchers and technical administrative personnel. The size of the WPs varied according to the available funding.

In addition, of the 27 stakeholders and external partners that were contacted, eight answered the online questionnaire. Five had OH project experience over the previous 10 years, one had short-term experience (between 1 and 5 years) and two had no prior experience in OH before UC-CARE.

Stakeholders and external partners stated they were mainly contacted by the PI at the beginning of the project and five of the eight declared they had provided direct scientific input to the project.

### Evaluation element 1: identification of the relevant context for the initiative

#### Description of the context

**Figure 1:**
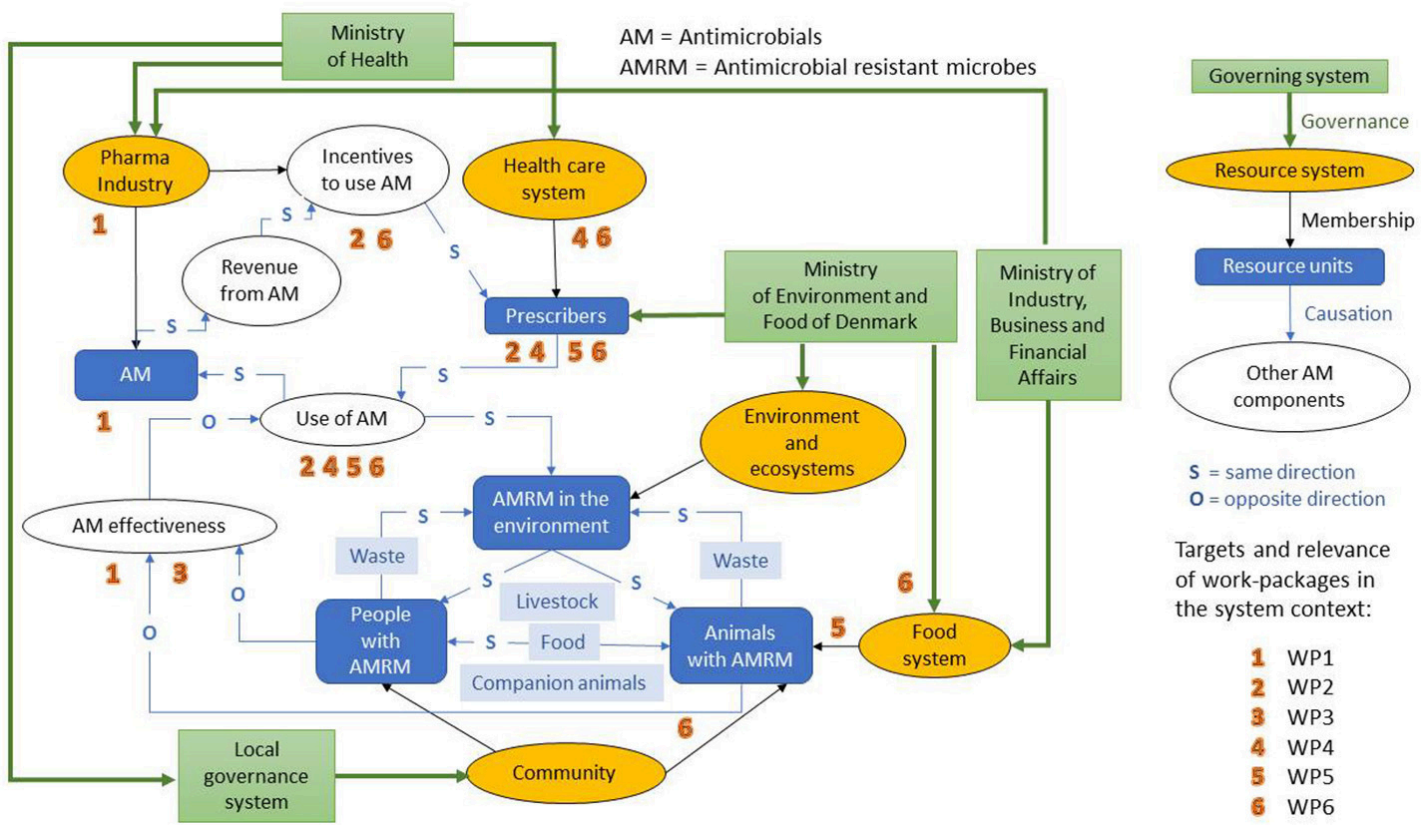
Visual representation of the context of AMR within Denmark relative to the UC-CARE research project, and an illustration of where UC-CARE work packages belong in the system. Work packages are described in the main manuscript text [Source: Modified from Rüegg et al. (26)].

The UC-CARE consortium operated within the overall context presented in Figure 1.

The overall **aim** of the system is to protect human and animal health in terms of AMR infections, as there has been an increase in deaths attributed to AMR worldwide (27, 28). Denmark has a highly organized and integrated surveillance, prevention and control system to minimize the AMR burdens in humans and animals through a process of science-based policy making. Reports are published by the different partners involved, and explain the annual outcomes and results, for example surveillance of antimicrobial use in animal production systems, health status of the country, reports about AMR cases, and final reports about relevant research projects initiated by government-supported research institutions. These reports are publically available and presented at public seminars, allowing change to be observed and implemented depending on the outcomes of the system, situation and public concern. The most comprehensive and consistent sources of information about AMR in humans and animals in Denmark, as well as the use of antimicrobials in animals, are the annual DANMAP reports (available at www.danmap.org, accessed on 26 July 2018).

As represented in Figure 1, **actors** in the system are located in the ministries of health, agriculture, environment and commerce, the health care system (including hospitals), pharmaceutical industry, food and agriculture industry, private practices for animal and human medicine, and research and education institutions. The relationships among actors are represented in Figure 1. Weighted links were not considered in the figure. Changing legislation and providing research grants should alter the activities of the different actors. Depending on the previous results of the system, actors should be able to react and modify their behavior and activities.

The **stakeholders** are also represented in Figure 1: community, patients, future actors (e.g., students). If the system produces the expected outcomes, the stakeholders should be directly affected by the improved health and reduced risk of resistant infections in both humans and animals. Changes from stakeholders can also be expected after suitable awareness campaigns, e.g., appropriate use of antimicrobial therapy from patients.

The main **dimensions of the system** include geographical, temporal, political, and legislative, as well as dimension of life, network and economy.

- **The geographical dimension:** the AMR threat is a worldwide public health issue, and any improvement would be valuable in every country. However, the system of immediate relevance to UC-CARE was the Danish AMR context and the impact on the human and animal populations of Denmark.
- **The time dimension** of the system is mainly based on years. Any changes within the system, e.g., developing and applying new legislation, application, and use of research results would require several years. Moreover, many research projects are executed on an annual or multiple-year basis. The system has no time limit, i.e., as long as the final objective has not been reached, i.e., as long as AMR continues to be a health threat, the system will remain.
- **The political dimension** is significant within the system. Several ministries are direct actors; industries are part of the decision and possible change within the system. Moreover, stakeholders such as the community are more and more concerned about AMR and the consequences for their health. The political dimension is limited by economic questions (e.g., costs of research, burden of the disease), public health issues (e.g., health threat, special care needed for infected patients) and progress of the research (e.g., discovery of new drugs).
- **The legislative dimension** is also significant in the system. The Danish legislation is of course the first step, but European and international legislation can also affect the system. Legislation provides a framework within which people and organizations can add their own decisions and standards to improve health for everyone. Results from research projects, drug discovery or the participation of researchers in evidence-based decision making could influence the legislative framework of the system.
- **The economy is also an important dimension** of the system. Costs and benefits are of concern to people and organizations at all levels, possibly limiting the progress and implementation of new discoveries.
- Also, **several** levels of the **dimension of life** are included in the system such as cell, wild and domesticated animals, individual humans, plant life, and the interaction of the human and animal populations. The network and the links between people and organizations are therefore a significant dimension of the system as they dictate the impact across the wider environment of animals and plants, as well as the human population.

#### The OH initiative UC-CARE within the system

The initiative gathered 14 departments from four different faculties of the University of Copenhagen, aimed at different aspects of the fight against AMR. Different disciplines were integrated in the initiative through veterinarians, microbiologists, economists, vaccinologists, pharmacologists, chemists, sociologists, psychologists, and physicians. Multiple sectors were targeted through the research, which aimed to: develop new antimicrobials, improve antimicrobial effectiveness, understand the mechanism of AMR, develop alternatives to antimicrobials (including vaccines and estimating their costs) and understand the drivers for prescribing antimicrobials. Human and animal sectors are commonly targeted. UC-CARE had six different WPs acting at different levels (Figure 1): WP1—drug discovery and translational research, WP2—alternative control strategies with focus on vaccine development, WP3—optimization of antibiotic therapy, WP4—comparison of current practices in human and veterinary medicine aimed at improving diagnostics, WP5—cost-benefit analysis and effects of management-based control options in livestock and WP6—societal issues and governance.

The initiative aimed to have an “impact on the life of humans and animals by providing new knowledge and solutions for enhanced diagnostics and antibiotic therapy of bacterial infections, leading to a significant reduction in the use of antibiotics in human and veterinary medicine” (extract from UC-CARE research funding proposal). The OH approach was integrated into the initiative at the proposal writing phase. It became apparent from the beginning that interdisciplinarity should be considered when deciding who should be involved in the initiative. Disciplines, hierarchies and responsibilities were shared among WPs from the beginning of the project.

**Actors** were from the University of Copenhagen, and included professors, PhD students, post-docs and other scientific and technical/administrative personnel. They were all located geographically close to each other as many were based in Copenhagen.

**Stakeholders** other than the actors were involved from the onset of the project, which aimed to include all possible partners from human and veterinary public institutions and private organizations. They were identified through the consortium (cf. OH sharing), and some were even more integrated into the process by participating in the research, being co-supervisors of students. They were referred to as “external partners,” and they could have an impact on the research and its development.

No legal **restrictions** were identified. The discovery of new drugs is not subject to regulation, only the market release, so no particular factor limited the initiative. Sociologists, psychologists, linguists, and economists included in the project aimed to identify any social restrictions among antimicrobial prescribers, patients, and animal owners in order to understand the drivers of antimicrobial use and any alternatives. In addition, the social stigmatization of people and owners of farms with AMR infections was investigated.

Some **consequences** or **impacts** of this initiative were expected at societal level, although it was hard to infer before initiation of the project which were most likely to be realized. For instance, some decisive findings could lead to other research projects and the future development of new drugs or vaccines, and the consortium could be expected to have an impact on decision making through engagement with stakeholders.

**Figure 2:**
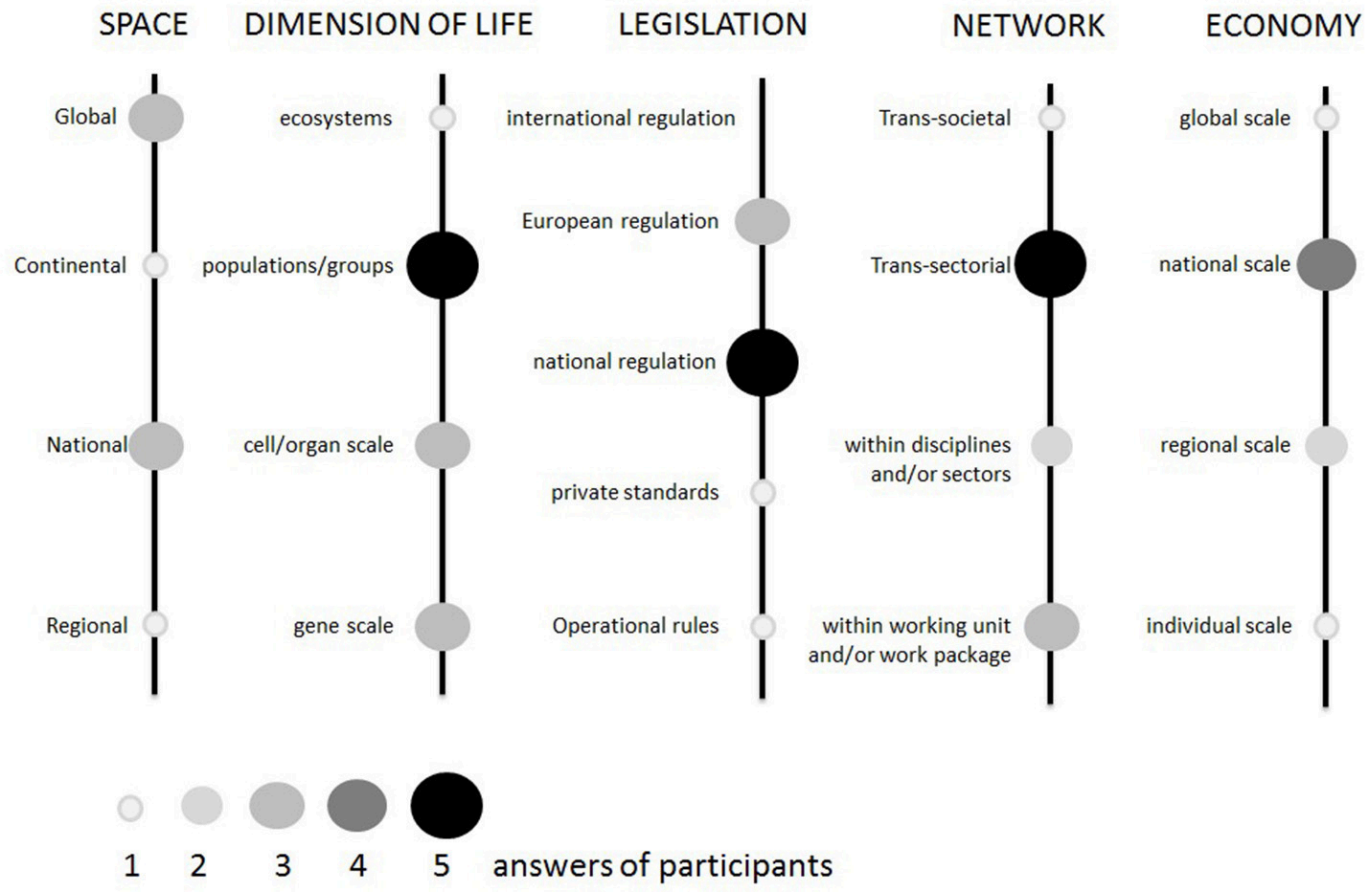
Answers from eight stakeholders and external partners to the online question, “how are the following dimensions (or aspects) reflected in the UC-CARE project outcomes? (Or how comprehensive is the project with regard to these dimensions of One Health?)”.

The main **dimensions relevant to the initiative** were the geographical, temporal, and legislative dimension, as well as the dimension of life, network and economy (i.e., most of the dimensions identified for the underlying system). When describing the dimensions reflected in UC-CARE outcomes, all stakeholders that responded to the online questionnaire had quite a global and holistic point of view (Figure 2).

### Evaluation element 2: theory of change of the initiative

**Figure 3:**
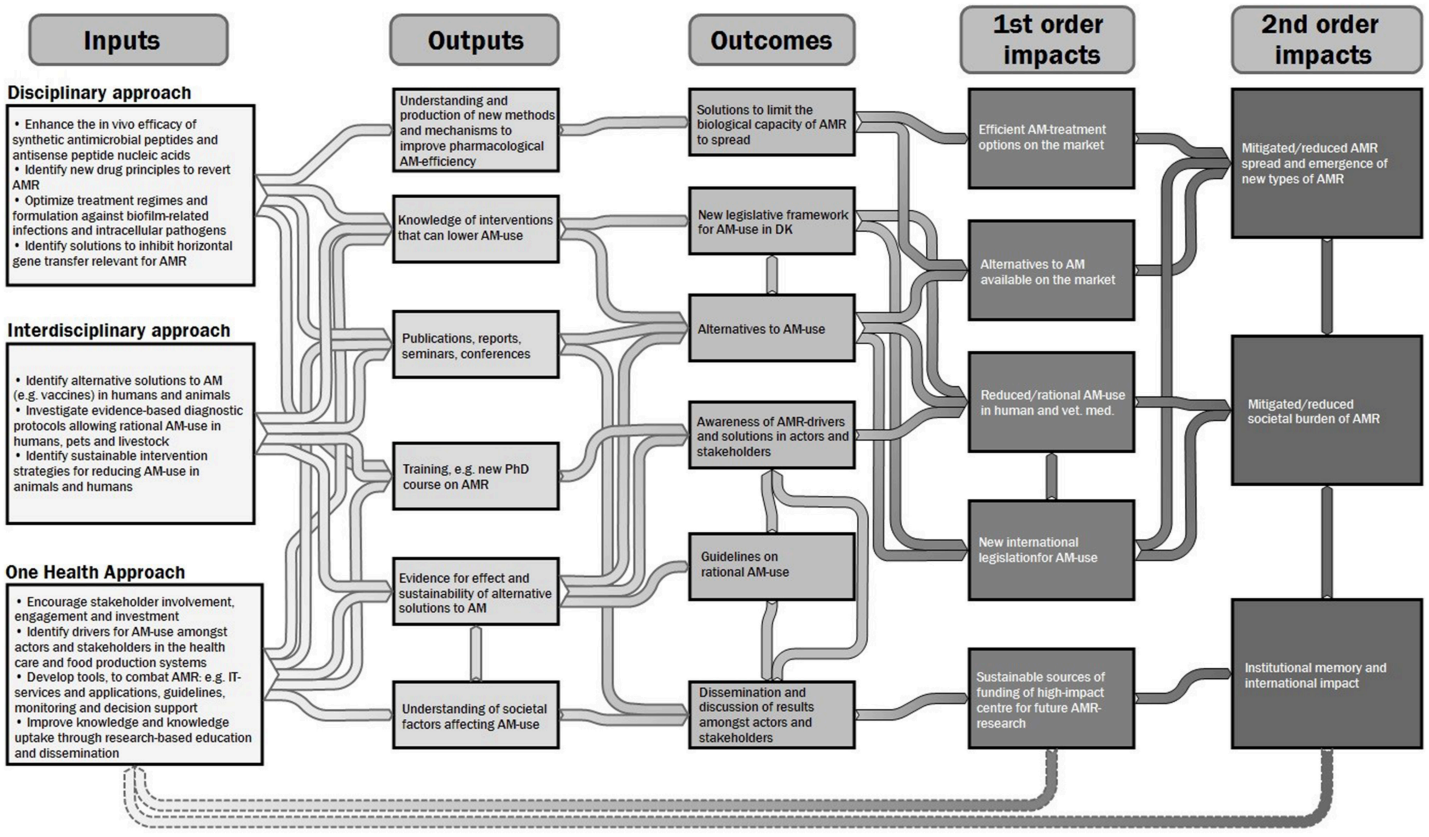
Authors' illustration and summary of the theory of change underlying the UC-CARE initiative. The information was mainly derived from the UC-CARE funding proposal and interviews with actors in the initiative. The figure shows the tentative links between inputs, outputs, outcomes and impacts (authors' analysis).

The TOC of the UC-CARE project is presented in Figure 3, and represents the different societal impacts that the initiative aimed for, together with the expected outcomes and the outputs determined by the different inputs and the linkages between these. Actions and inputs of the initiative were based on different sources and resources, such as results from previous studies, available Danish data, interviews with actors at governmental and societal level and the participation of patients, animal owners and prescribers in several studies.

### Evaluation element 3: one health-ness evaluation of the initiative

#### Degree of one health-ness

The NEOH tools were completed based on the responses to the online survey and face-to-face interviews, as well as the information provided in the consortium proposal and mid-term evaluation report. The most relevant outcomes of the interviews are detailed below and the completed assessment table is available in Annex 3 (Supplementary Material).

The UC-CARE was a 4-year project and was therefore limited in time relative to the system. Moreover, the project was focused on the Danish system: all researchers were based in Copenhagen and some WPs focused on the particularities of the Danish system (e.g., cost-benefit analysis, effects of changed legislation, treatment guidelines for antimicrobial use). Other WPs worked across borders (e.g., prescription practices and perceptions of doctors in Denmark, France and Italy) and some of the technical results (e.g., within drug development) are of international relevance. Therefore, several outputs might still have an impact at European and international levels for years after the finalization of the project.

The main tool for communication among all participants was the annual consortium meeting. The meetings were the only opportunity to gather all participants, external partners, stakeholders, and advisory board members. The only other consortium-based support for communication and exchange of progress, results and updates among the participants and WPs was the International Conference on One Health and Antimicrobial Resistance (ICOHAR), organized by several of the consortium members and held from 30 September to 2 October 2015. During these events, the consortium partners had the opportunity to discuss and gain a better understanding and a more global overview of the topic of AMR and its issues. All interviewees mentioned that they gained a more comprehensive way of thinking about the AMR challenge and an understanding that all actors were involved and responsible. The annual consortium meetings were described by some interviewees as “generally interesting,” but also as “very superficial” and a “political aspect of the project.” Some compared it to reading a newspaper: “really interesting to hear about new things, but forgot them right after the meeting.” Others were less impressed and described the meetings as “horrible” and “incoherent” with no real interaction between people, and not very informative. All interviewees mentioned that the difficulties experienced with communication during the annual meetings were probably linked to the major issue of not speaking a common scientific language across disciplines, and a lack of focus on making the methods and results understandable for everyone.

WP6 on “societal issue and governance,” and to some extent WP5 on “cost-benefit analysis” were mentioned by participants from other WPs as not being easily integrated into the discussions. Some interviewees had the perception that the economists and sociologists did not understand the research at a cellular level and became somehow excluded from discussions, becoming less and less involved in both the discussions and meetings. Interestingly, the sociologists and economists did not share this perception. Also worth noting is that all participants from other WPs indicated that results from WP5 and WP6 were really interesting.

Interaction with and interest from stakeholders varied a great deal among the WPs: from external and strong partners being directly involved, to limited contacts. Several interviewees mentioned that the impact of stakeholders was low, and their participation in the annual consortium meetings was described as decreasing over the duration of the project. The meeting took place once a year and included an exchange of results, but stakeholders' input was limited. Some interviewees mentioned that the project could not be completely transparent with the stakeholders, and that it was not possible to share data with them. They had limited exchanges because the project researchers had to “keep their cards close,” and the ownership of data was a major issue, with intellectual rights/technology transfer departments on both sides drastically slowing possible exchanges of data. This could explain the limited interactions with companies. One WP organized an exchange with a diagnostic company for PhD training. However, one WP reported sharing of data with stakeholders without any issues. No stakeholders from the general public (e.g., consumers or animal owners) were involved in the project except as study participants or animal owners in some of the observational studies in WP4, WP5, and WP6.

Differences were highlighted among the WPs in their global organization and the application of OH. This clearly led to different experiences of the OH approach among the participants. Discussions and knowledge sharing were usually organized among the different disciplines within the WPs to discuss progress and methodology. The WP4 “mapping of current practice in human and veterinary medicine” was mentioned four times by interviewees as the “real” OH WP of the project, with an advanced joint research among different sectors and disciplines. Three interviewees mentioned that the project was mainly focused on PhD students and post-docs, and that it was difficult to maintain interdisciplinarity because PhD students and post-docs need to focus on a particular topic to reach a necessary level in their field. The perception seemed to be that completely integrated, interdisciplinary research would be more easily driven by more experienced researchers.

Collaboration was mentioned among some WPs, but mainly between researchers from closely related disciplines. Interviewees also mentioned regular collaborations with scientific partners outside the project. This could include researchers from the same university or foreign partners.

Two interviewees mentioned that the project mainly gathered microbiologists, and that this could be related to the PI being a microbiologist. They suggested that while writing the proposal, the initial PI could have been more inclined to write it from a microbiologist point of view. Other interviewees mentioned that medical and veterinary science disciplines represented 80% of the actors, whereas e.g., chemists were less well-represented within the project. Those assumptions could not be confirmed as no all-inclusive list of UC-CARE participants was available to the evaluators.

The deficiencies identified in the working characteristics related to gender imbalance (i.e., male domination) and a disparity in the representation of different disciplines, which could be partly due to a lack of open-mindedness toward other disciplines and sectors. We also identified a lack of mid- and long-term flexibility due to fixed research objectives for PhDs and post-docs, as well as a lack of regular collaboration among the different units in the consortium. UC-CARE did not manage to allocate resources for internal communication processes and tools, nor to generate the planned IT-tools dedicated to monitoring and decision support for patients/farmers and doctors/veterinarians and for communication and mediating technologies.

**Figure 4:**
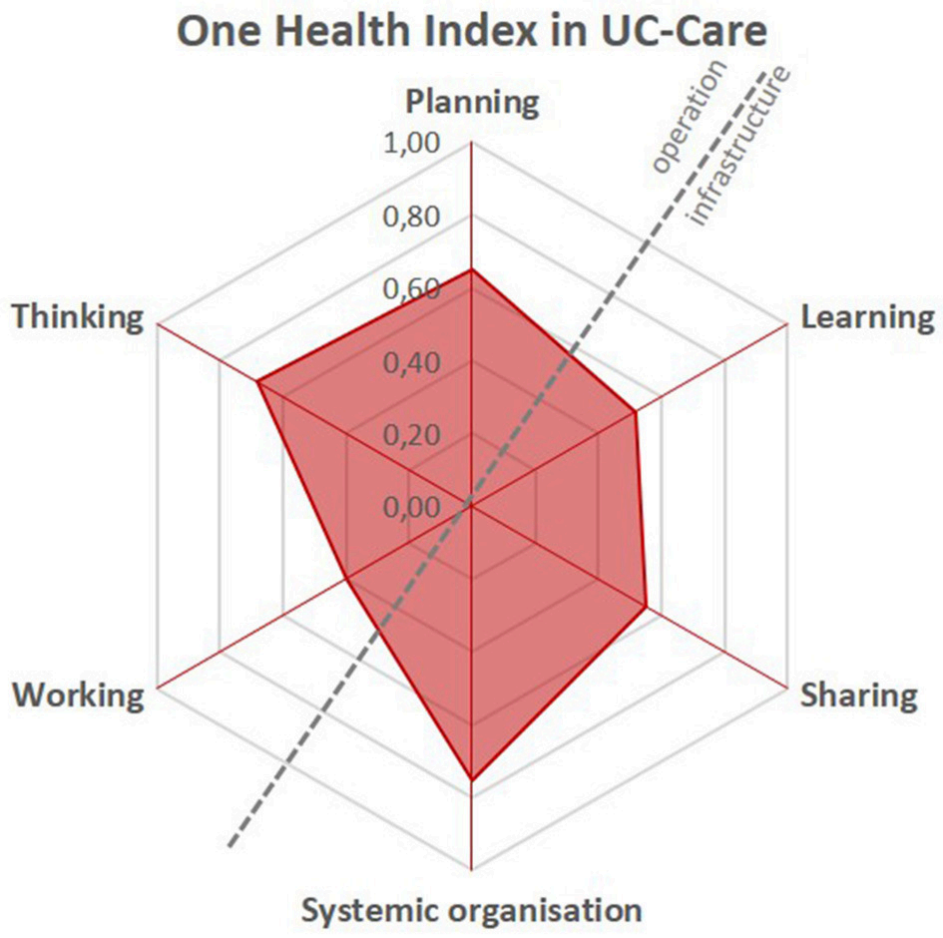
Spider diagram representing the scores allocated to the elements Thinking, Planning, Working, Learning, Sharing and Systemic organization of the OH-ness assessment of UC-CARE on a scale from 0 to 1 for each element.

In summary (Figure 4), the project was well-intended and thought through, as shown by the relatively high scores for planning, thinking and systemic organization. The project leaders' understanding was global and integrated all important aspects of OH. However, implementation during the project period was more difficult, particularly in terms of working and sharing, which can eventually also affect learning across such a consortium. This fact was mentioned several times by the participants and is reflected in the low overall scores on working, sharing and learning.

The OH index of UC-CARE was 0.34, which according to the NEOH framework can be interpreted as a mediocre level of implementation of an OH approach in the initiative. The OH ratio was 1.1, indicating a balance (close to 1) between the OH operations and supporting infrastructures. However, the ratio does not say whether these were then prioritized sufficiently. The formulae for calculating the index and ratio are provided in the introduction paper by Rüegg et al. (26) and in the OH Index Ratio sheet in Annex 3 (Supplementary Material).

#### Pros and cons of the OH initiative implementation

The UC-CARE participants stated that the main advantage of the consortium was its ability to broaden their interest in other disciplines and methodologies. They also mentioned that the project increased and improved their networks, which in some cases had led to new partnerships and research applications that would not have been realized or would have been constructed differently without the consortium. During group interviews with PhD students and post-docs, all interviewees mentioned they would search for OH working roles in the future, if possible. However, two interviewees clearly mentioned that it was not a main driver for their future jobs in research.

Two interviewees mentioned that interdisciplinarity in research was difficult because publishing together was a challenge. Some papers were written with authorship from different disciplines, but publishing a single discipline-targeted paper was found to be less challenging.

#### Critical review of the initiative in relation to an OH approach

All interviewees mentioned that this project and this new approach changed their mentality about other disciplines and even about the AMR challenge. They acknowledged that their understanding of the issue on a global scale had improved during the project. Interest in other disciplines grew among all interviewees, but for most of them it was not highly influential in their daily work. However, they all mentioned that the OH approach could have been pushed further in UC-CARE, and that it was not fully pursued due to a lack of experience with the OH approach.

Moreover, several WP leaders mentioned that the OH approach did not reach the people working in the laboratories, but was instead mostly perceived at senior/WP leader level. This was confirmed by the early-career researchers, who explained that they were not previously aware of OH. They learnt about this new approach and about working together in an interdisciplinary environment through the project. However, the interviewed early-career researchers did not have the opportunity to experience multi- or interdisciplinarity in their daily work.

Interviewees stated that they sometimes developed ideas and learnt lessons from this first OH initiative to establish better links among research WPs and disciplines. Some stated that more funding would have allowed larger teams (hence with a larger impact) with more legitimacy to spend time on collaborations and interdisciplinary work, and with someone in charge of coordinating joint work. The PI's commitment was not considered sufficient to sustain an OH approach in this consortium; a greater investment of resources would be needed to promote more transdisciplinary tasks. In addition, the WPs could have been organized differently, with continuous and common goals identified. An interviewee mentioned that co-supervision of PhD fellows within and between WPs would support interdisciplinary research.

**Figure 5:**
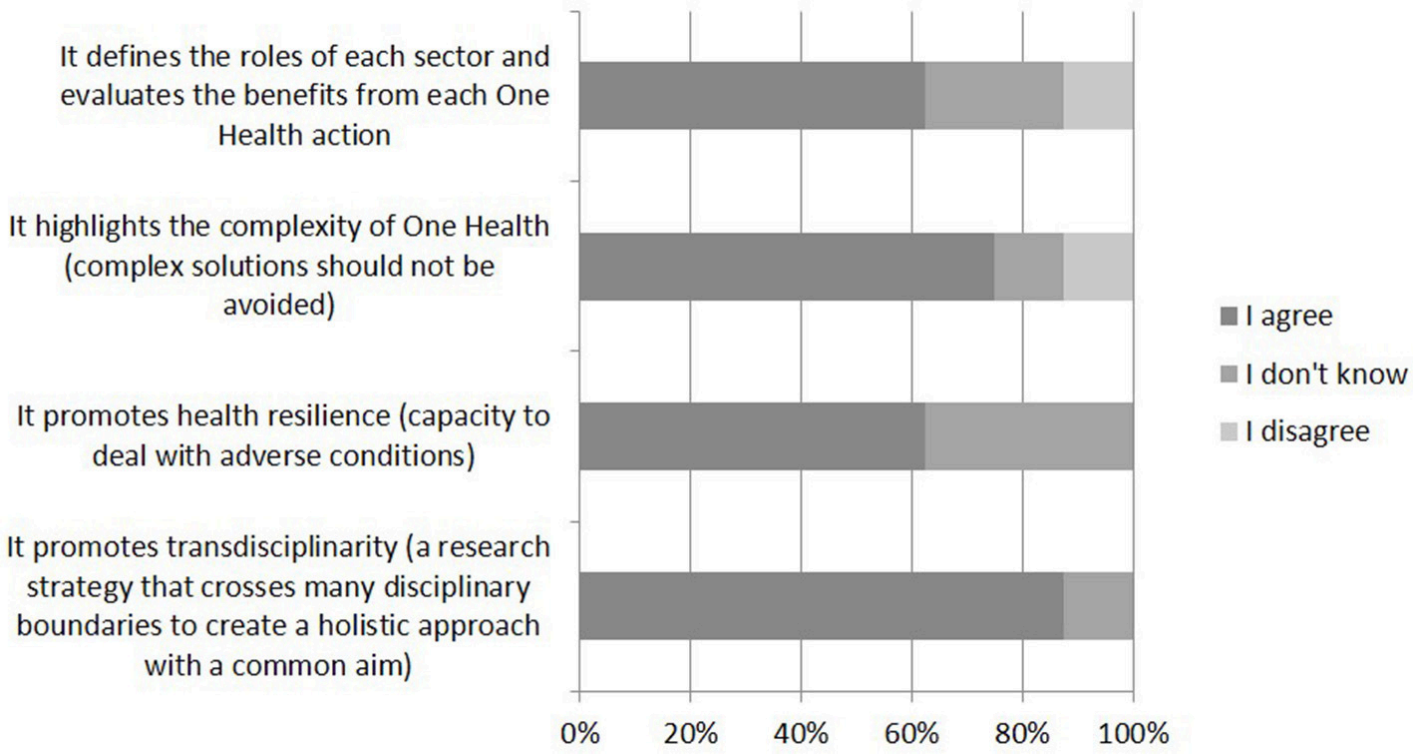
Answers from eight stakeholders and external partners to the online question, “which of the following do you see as benefits of the One Health approach?”.

Stakeholders and external partners reported having a good understanding of OH and its importance (Figure 5), with all agreeing that an OH approach is relevant to the AMR topic. However, the application of an OH approach was more difficult because more time was required for it to be realized and because the workload did not appear to be truly balanced among disciplines. The stakeholders reported that the final results were less remarkable than they had expected. However, they also indicated that UC-CARE seemed to be a good experience that should be extended and developed.

### Evaluation element 4: outcomes

Numerous and diverse outputs and outcomes were identified in UC-CARE and these are detailed in Table 1.

**Table 1:** List of different outputs and outcomes of the UC-CARE project for the categories: disciplinary, interdisciplinary, OH and unexpected outcomes and outputs.

Disciplinary outcomes and outputs	- A large number of scientific papers - PhD theses - Department/university recognition at international level - Development of new networks and projects - New high-profile funding for a long-term research efforts based on UC-CARE - New funding for long-term research efforts based on UC-CARE
Interdisciplinary outcomes and outputs	- Some scientific papers - Development of new networks and projects - Interest of participants in interdisciplinary work and results - Treatment guidelines for antimicrobial use for humans and animals
OH outcomes and outputs	- OH courses at DK university for PhD students and post-docs - Common course for human and veterinary medicine candidate students - Interest of participants in OH approaches - Learning/understanding of the planning of organization and resources in OH consortia
Unexpected outcomes and outputs	- New experience that it was difficult to plan, perform and report interdisciplinary research - New national Danish legislation with direct reference to UC-CARE results - Initiation of treatment guidelines for antimicrobial use at EU level - Three seats in the National Council for Antimicrobial Resistance - One seat in the Council for Improved Hygiene

The UC-CARE proposal included the objective to provide “new knowledge and solutions.” It was expected that OH outputs and outcomes would have an impact on human and animal populations through new knowledge and guidelines for the prudent use of antimicrobial. It was also expected that the actors would produce many types of publications and that disciplines would learn from each other and start up new projects and collaborations. Finally, new educational activities created in collaboration among multiple disciplines were planned at PhD level (Table 1).

The final number of publications will not be known until the final UC-CARE report becomes available during 2018, as no central collection of publications is currently available. However, according to the mid-term report that was sent in for evaluation by an external panel in April 2016, 33 international peer-reviewed journal papers had been published, 13 more submitted and 25 papers were listed as being in the pipeline. Most of these were outputs of specific disciplines, but it was noted in the report that an additional 25 publications were anticipated in the UC-CARE consortium, many of which would be authored by interdisciplinary teams. As mentioned by the interviewees, it seems that it typically takes longer to write and publish papers from interdisciplinary teams. The fact that it was difficult to extract information about the output of the consortium in terms of publications is reflected in the relatively low scores for sharing and learning in the NEOH framework. Structures to improve knowledge and information sharing across OH consortia should be considered. These could include online resources, newsletters explaining new results, transdisciplinary research activities, interdependent tasks and more joint teaching activities among the WPs.

Several points were highlighted by participants throughout the interviews. Participants clearly indicated that they achieved new knowledge and solutions in their own research field. The problem of AMR had been understood a bit more, allowing going further in terms of improving health. Some results could even have a very high impact, yet there tended to be a gap between the results and their applicability that would need several further steps. Some results, however, could already be applied at the time of the evaluation. One interviewee mentioned that results were important but, in a project such as UC-CARE, outcomes had to be explored and thought of differently and in broader terms. They explained that when looking from a different perspective the success of the project could be attenuated. Future collaborations were agreed and the money was fairly well-spent, yet they thought that disciplines could have provided more “strict” science results. Their definition of “strict” scientific results indicated a paradigm of hypothesis-led research with little understanding of how the hypotheses were originally framed and constructed, and which is largely driven by previous work and available measurement tools. They admitted that UC-CARE involved many scientific disciplines, which contributed greatly to the common goal.

## Discussion

### Discussion of evaluation methodology

The evaluation process as defined by NEOH generally went well and was positively received by the participants. Those who were contacted showed a general willingness to participate and to be interviewed. However, many early-career researchers were difficult to reach or replied that they had left the consortium after finalizing their project activities, while some never answered. The reasons for these difficulties relate to the increased workload of some individuals as their PhD projects came to an end, and some being on maternity leave and/or having already left the project. The PI and one internal partner supported formally the evaluation among the consortium (e.g., sending emails). That was beneficial and probably encouraged participation. Despite these difficulties, the study is based on a reasonable number of interviews that overall were representative of the broad range of disciplines in the UC-CARE consortium.

As expected, data collection was the most critical and time-consuming part of the evaluation. The NEOH tools require a large amount of data and information, and as with any thorough evaluation process, this can raise difficulties. Data were not always available from written internal documents and had to be supplemented with interviews, providing potentially subjective information. As the data needed were unusual and differed from other types of research evaluations, the questions sometimes puzzled the interviewees, yet they all made the effort to answer the questions and rethink their project activities. The semi-open interview format allowed information that was not predetermined to be gathered. The online questionnaire for the external partners and stakeholders was less successful (only eight answers out of 27 people contacted), which could be due to: (i) the length of the questionnaire, (ii) some stakeholders no longer being involved in the project (e.g., change of employment), and/or (iii) some stakeholders having little involvement in the project from the beginning, thus feeling that they did not have much to contribute.

The first steps of the evaluation (i.e., context description and TOC) were descriptive and required a global and detailed understanding of the context, the initiative and a deep reading of all internal documents such as the project proposal and mid-term evaluation report. Ideally, this exercise should have been conducted by the project initiators during the proposal writing phase. The presentation of the results at the annual meeting helped gather comments and remarks about the description and understanding for the TOC elaboration. The completeness of the TOC reflects the ideas of the UC-CARE participants and could overlook important elements such as unwanted side effects of outcomes (e.g., a discovery of new drugs could lead to new types of or more AMR) or other unexpected outcomes (e.g., durable impact of knowledge gained through courses among PhD students and the scientific community) (29). Durable feedback loops in the TOC were not identified or anticipated in the proposal. The project would probably have benefited from more consideration about the logics in the TOC in advance. Participants and partners would have been able to better understand the expected changes in the context and to identify the OH outcomes. To assist evaluators of OH initiatives, the authors recommended that NEOH elaborated on proper TOC descriptions and ways to link these with the OH-ness evaluation and the expected and unexpected outcomes in the overall evaluation in their handbook of OH evaluation.

The evaluation was mainly conducted by two assessors, but this was counterbalanced by the global review of the evaluation by two other authors and a discussion of the results with the UC-CARE consortium members. Two of the authors were internal actors and two were external to UC-CARE. The knowledge that the internal actors had about UC-CARE was very valuable to understanding the global functioning and processes of the research project, but external and internal evaluators will typically have a complementary overview of the context and initiative (30). Integrating internal partners can be a challenge as they can have a biased understanding of the initiative. However, total objectivity can never be reached (31). By integrating internal and external evaluators, we hoped for a balanced and neutral approach in the evaluation. Moreover, due to the complexity of OH challenges, the evaluation of OH initiatives and their outcomes cannot be expected to be an intuitive and easy task (15, 32), and combining the strengths of several evaluators should be an advantage.

### Discussion of results

As described above, the mid-term evaluation of UC-CARE deemed the project successful, based mainly on traditional research evaluation criteria focusing on the number and impact of publications and disciplinary research outputs, successful pursuit of PhD and post-doc tasks and dissemination and uptake of research results in the pharmaceutical industry (24). The present study provided different and complementary insights into the underlying operations and supporting infrastructures of the UC-CARE consortium and research project, and allowed us to capture its complexity and OH approach in more detail. The OH evaluation should be valuable for the consortium as it is based on a broader range of parameters relevant to the societal impact of the initiative, and highlights other strengths, weaknesses and lessons learnt by the use of an OH approach for AMR-related challenges than traditional research evaluation methods. In the authors' view, the results of the evaluation could also be useful for future proposals by research consortia keen to build their projects on an OH approach.

Interestingly, the shortcomings identified in UC-CARE were to some extent similar to those found in an evaluation of an OH surveillance system for West Nile Fever bringing together public health, veterinary public health and entomology experts (11). Indeed, working and learning characteristics were also scored lowest in the West Nile Fever-initiative albeit higher than for UC-CARE. We also identified a lack of mid- and long-term flexibility, planning processes, and resources dedicated to sharing. This is unfortunate as it has previously been shown that societal learning is important for control strategies to have an effect (33). In other words, although the idea behind UC-CARE reflected in the TOC was highly relevant, reasonably well-planned before the initiative and seemed highly integrated with many disciplines and with relevant stakeholders involved from the beginning, it proved difficult to carry out the OH approach in practice, and many of the actors went back to uni-sectorial and disciplinary work in their daily tasks. This was to the benefit of the disciplinary outputs, but potentially reduced the societal impact of the initiative.

Also, participants mentioned several times that it was difficult to publish interdisciplinary papers. Researchers and research projects are usually evaluated by the disciplinary quality and impact of their publications, whereas the OH approach has not been perceived and promoted as a quality characteristic in journal papers to date. Early-career researchers were particularly concerned about this, and the issue is underpinned by journals targeting OH issues being ranked low on impact, see e.g., One Health: https://www.journals.elsevier.com/one-health (accessed on 26 July 2018).

The low scores obtained for working, learning and sharing can be explained by several factors. The project was a first attempt at a large OH project in this organization, so there was little experience to build on. Due to the funding framework, the project promoted young researchers, who must focus on particular disciplines in order to become specialized. This refers the issue back to funding bodies, who must appreciate that the impact of research is influenced by additional means other than publication metrics, while scientists tend to base their activities on criteria and indicators that are applied in evaluations (34).

Changes in the mentality of the scientific community would promote OH by, for example, defining interdisciplinarity as a specialization. However, thinking, planning, and systemic organization received high scores, and the project was prepared and built in a clear and conscientiously way by the first researchers involved in the proposal, who all seemed to have a good understanding of OH approaches. Interviewees mentioned several ideas to improve the quality of the OH approach, e.g., allocating resources differently and defining a specific budget for OH (including collaboration, engagement of the public stakeholders, coordination promoting new collaborations across sectors) that could be used throughout the project. In addition, the WPs could be organized differently, to encourage more interaction among the different disciplines. The supervision of PhD students could be shared among different disciplines and could involve more senior researchers who could dedicate part of their time to interdisciplinary activities and allocate a larger budget so that more laboratory personnel could be involved. The project could also benefit from having a budget for a specific OH coordinator in the project, whose role might include promoting the exchange of information and results among center members, organizing workshops and learning activities, and developing the relationship with stakeholders. Importantly, limitations in the project did not reduce the motivation of partners in pursuing OH projects in the future.

It should be noted that the OH index and ratio are single numbers that cannot reflect the variation among actors in terms of their personal experience. Indeed, each UC-CARE member experienced the initiative differently according to their experience, the WPs and their personal interest. The index and ratio might eventually be compared across OH initiatives to assess whether it is important to score certain characteristics higher than others to promote health-improving societal changes, e.g., sharing and learning, as suggested above.

Differences were identified among the WPs in UC-CARE. For example, WP4 was already organized to provide an interdisciplinary environment to compare human and veterinary medicine practices. All interviewees of WP4 were enthusiastic about their experience and acknowledged that working in this interdisciplinary environment improved the quality of their work and outputs. Differences in experiencing OH were also seen at an individual level. Some interviewees were more reluctant and disparaging of their experience than others. Conducting several interviews for each target group allowed us to gain a better understanding of the real situation, and highlighted the potential differences between WPs and individuals.

UC-CARE involved the different stakeholders in the process from an early point. This is not unusual for such projects, but it indicates the interest of UC-CARE in providing useful results. Some partners worked closely with PhD students, allowing the exchange of data and discussions concerning the progress of research. This aspect is expected to be important and valuable for the project (35). The process of stakeholder selection was not systematic, but no major gaps were identified. This may be attributed to experienced researchers bringing their trusted networks into the consortium; an important quality to acknowledge.

Finally, noteworthy positive mental and structural changes and improvements were consistently identified across interviews. For example, all actors mentioned a new interest in the disciplines and work of others, and in future joint proposals, as well as expanded networks. They all acknowledged the necessity for and benefit of working with other disciplines, and from this point of view, the initiative was a success. In addition, the outcomes identified during the evaluation (Table 1) can be expected to have an impact on AMR development, and unexpected outcomes were identified, which led to changes in legislation and strengthened representation in advisory forums, which may in turn lead to more evidence-based policy development. The latter is indicative of the lasting impact of the initiative on AMR policy, which can be considered the ultimate goal of research conducted with public funding.

## Author contributions

AL and LN were substantially involved in all steps of the study from the conception to the design of the work. They also primarily drafted the paper and implemented several substantial contributions from other co-authors. KS and JR were also involved to some extent in these steps. Their contribution was valuable and essential to the final version of the paper. AL, NL, KS, and JR approved the final version of the paper, submitted to Frontiers. They also agreed to be accountable for all aspects of the work, in ensuring that questions related to the accuracy or integrity of any part of the work are appropriately investigated and resolved.

### Conflict of interest statement

The authors declare that the research was conducted in the absence of any commercial or financial relationships that could be construed as a potential conflict of interest.

## Footnotes

^1^https://www.frontiersin.org/research-topics/5479/concepts-and-experiences-in-framing-integration-and-evaluation-of-one-health-and-ecohealth.
