# Supplementary material for: A One Health Evaluation of the University of Copenhagen Research Centre for Control of Antibiotic Resistance
Source: Front Vet Sci. 2018 Aug 21;5:194. doi: 10.3389/fvets.2018.00194 (PMC6110841; doi:10.3389/fvets.2018.00194)
Supplement: Supplementary file 1 [file Table_1.DOCX]

Supplementary Material

A One Health evaluation of the University of Copenhagen Research Centre for Control of Antibiotic Resistance

**Anaïs Léger*, Katharina D.C. Stärk, Jonathan Rushton, Liza R. Nielsen**

*** Correspondence:** Anaïs Léger, anais.leger@safoso.ch

# Questionnaire for semi open interviews with UC-CARE actors

**Planning**

1. What are the different tasks planned in your WP? And then, considering each tasks:

- What are the stakeholders involved in the accomplishment of this task?
- What is the responsibility of each stakeholder?
- Describe the authority of each stakeholder in this task.
- What are the means needed to accomplish this tasks? Does it match with the reality?

1. Which stakeholders are concerned? State the groups, individuals and dimensions that are concerned by the problem.

- Has a process to identify and involve all essential stakeholders (including governmental, academia, industry, NGOs) been described and followed in the project?
- Have essential stakeholders (including governmental, academia, industry, NGOs) been involved at an appropriate level throughout the duration of the project?

1. Is the non-scientific community involved?
2. Are there powers (i.e. academic or disciplinary dominance) or gender imbalances within the group, which risk biasing the process?
3. Can you describe your team? E.g. number of people, meetings…

**Learning**

1. As an individual, which kind/level of information/knowledge did you receive within this project? How frequently did you receive information/knowledge from each category? and how frequently?
2. Within your work package, how do all the researchers exchange theirs ideas and information/knowledge? What is the level of discussions raised? and how often?
3. At project level, how is organized the transfer of information/knowledge? What is the purpose of this information/knowledge exchanges?
4. Direct environment (other entities in which the project interacts ex: competitors, customers, suppliers, strategic partners, distributors…) Is this direct environment supportive for leaning in your WP-project? At which level?
5. Indirect environment (nonspecific elements of the project’s surroundings that might affect its learning like economics, technological, socio-cultural and others) Is this indirect environment supportive for learning in the project?

**Sharing**

1. Sharing mechanisms

- Does the initiative have appropriate mechanisms in place to facilitate sharing of information within the initiative? (E.g. newsletters, workshops, reports available to all, results getting published, online information sharing platform…...)
- Does the initiative have appropriate mechanisms in place to facilitate sharing of information outside the initiative? (E.g. newsletters, workshops, reports available to all, results getting published, online information sharing platform…...)
- Have resources been allocated to ensure necessary data and information sharing?
- Have appropriate (e.g. formal/written/signed) agreements been made concerning data sharing in the initiative?
- Are mechanisms/procedures in place to ensure data quality, e.g. data completeness, error-checking and correction of errors, variable descriptions, description of aggregations/calculations, documentation available.
- Are mechanisms/procedures in place to ensure safe and appropriate data storage? (e.g. type of software, server, backup)
- Are mechanisms/procedures in place to ensure safe and appropriate data accessibility to facilitate sharing? (e.g. is extraction of data feasible without access to experts, or are experts readily available for extraction of data, is the process of data extraction bureaucratic/ cumbersome/overly time-consuming?)

1. Sharing functionality

- How well are data being shared between people within the initiative? (e.g. compartmentalised (score 0-33), shared between few groups (34-66), fully shared between all in the initiative (score 67-100).
- How well are methods shared between people within the initiative?(e.g. compartmentalised (score 0-33), shared between few groups (34-66), fully shared between all in the initiative (score 67-100).
- How are results shared between people within the initiative? (e.g. compartmentalised (score 0-33), shared between few groups (34-66), fully shared between all in the initiative (score 67-100).
- Sharing flexibility/future
- How well does the initiative include the creation of potential institutional knowledge reservoirs for data, methods and/or results over time?
- Are mechanisms/procedures in place to safe-guard data and information access in case of system change, e.g. change of IT-system, data ownership, institutional organisations.
- To which extend are data and information produced in the initiative used for learning/education activities?

**Working**

1. Is interdisciplinarity required to solve this problem?
2. What are the benefits of using interdisciplinarity rather than a conventional approach?
3. Is the problem relevant to the health of people, animals and/or the environment?
4. Is it relevant to One Health?
5. How diverse are the disciplines, methods, scales of analysis and/or social actors involved?
6. To what extent have the different disciplines worked together?
7. What is the spatial proximity among disciplines’ offices? Are there face-to-face interactions? If yes, how frequent are these interactions?

**Thinking**

Objective: score different dimension within the considered WP, some can be pertinent or not (can exclude them). The mentioned dimensions are: space, time, dimension of life, legislation, knowledge, management, network and economy.

1. Is this dimension relevant for your WP? For the whole project? Does it influence a lot your way to conduct your studies?
2. Do you think it’s an important criterion for your work? And how much?
3. How are these dimensions reflected in your project? How is OH reflected in your project?
4. How these dimensions are comprehensive/included within your project?

**Back to UC-Care proposal**

Time is ripe to address this important societal challenge in a more comprehensive and innovative way. Consequently, a group of expert researchers at UCPH has joined forces for the creation of UC-CARE, a One Health interdisciplinary and inter-sectorial research centre for control of antibiotic resistance. The aim of UC-CARE is to impact the life of humans and animals by providing new knowledge and solutions for enhanced diagnostics and antibiotic therapy of bacterial infections, leading to a significant reduction in the use of antibiotics in human and veterinary medicine.

1. Does the case study take up a One Health problem, and how is this problem relevant (to what?)
2. Is the One Health problem adequately translated into scientific questions? Is the current state of knowledge taken into consideration and what is innovative in relation to this state of knowledge?
3. Do the methods envisioned, the interfaces of transdisciplinary collaboration, the form of integration in practice, and the outcome of the case study fit the solution strategy sought for in One Health?
4. Did UC-CARE finally meet its objectives?

# Online survey for stakeholders and external partners

**
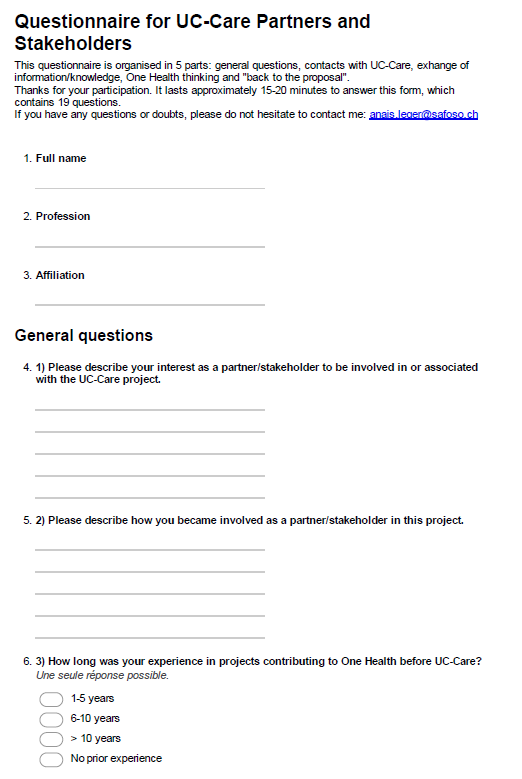
**


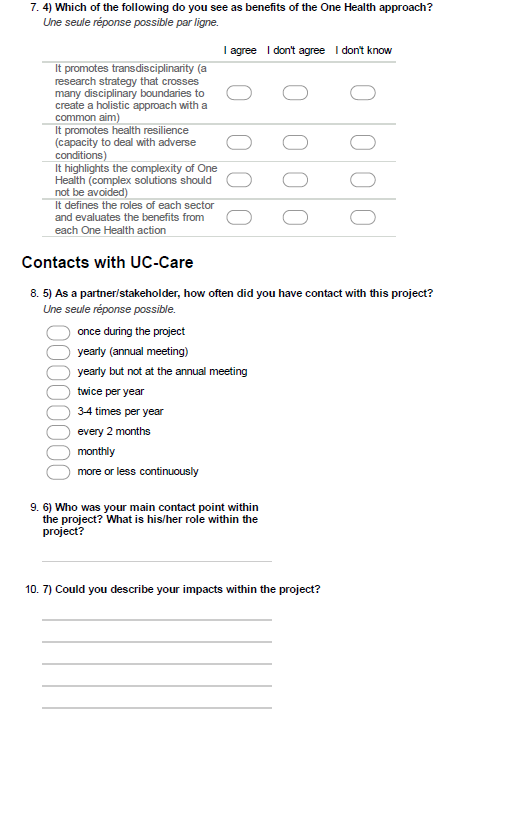

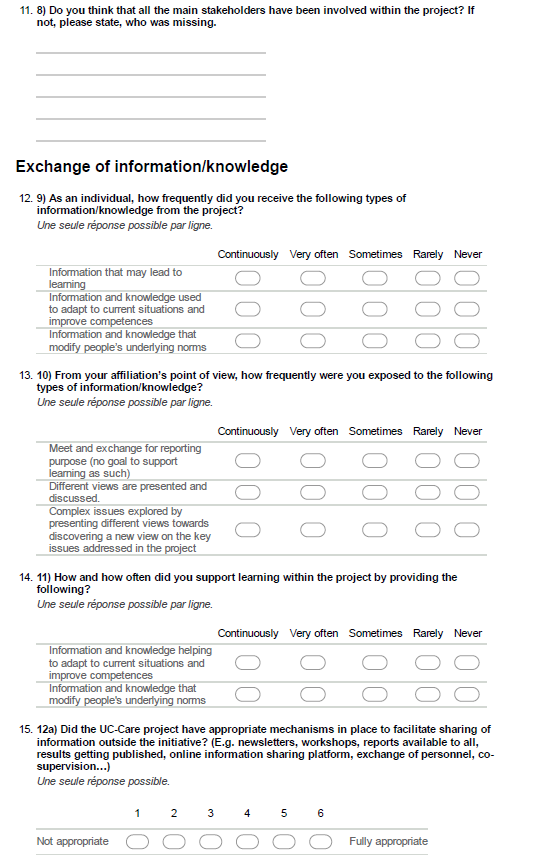

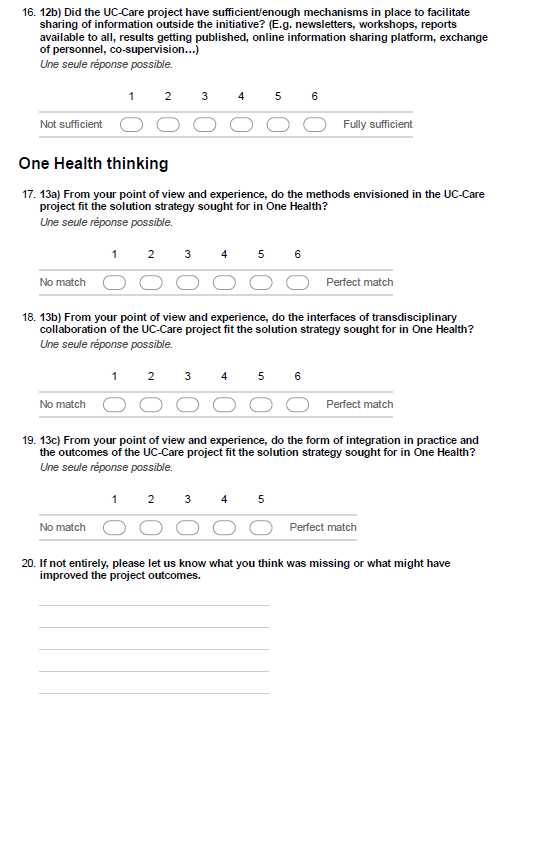

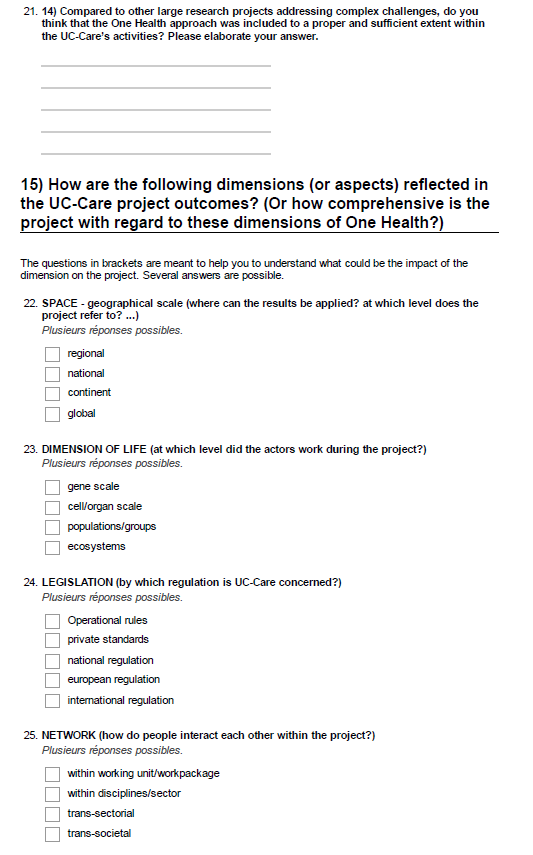

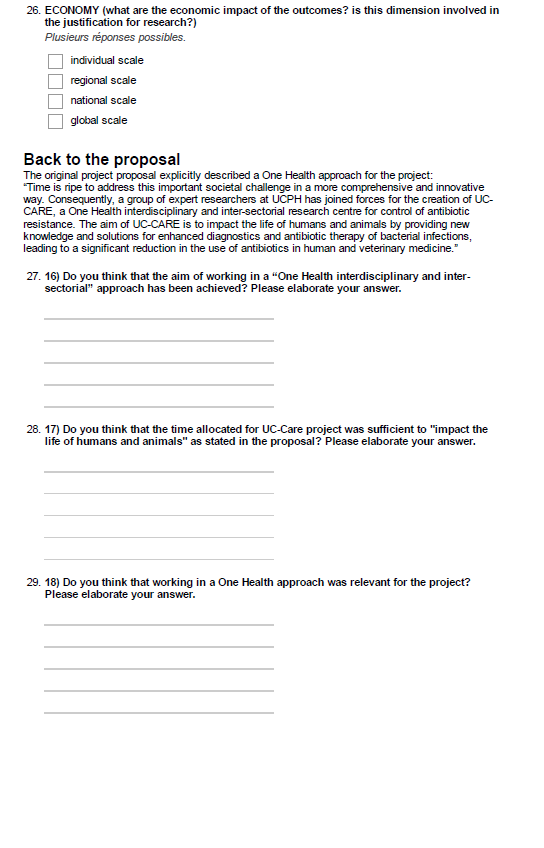

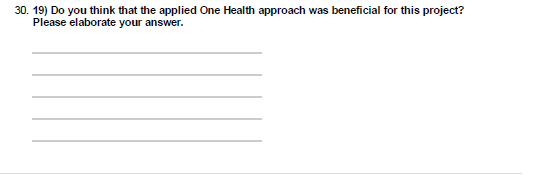


# Completed NEOH table for the assessment of the OH-ness of UC-CARE

Excel document (8 different sheets)
